# High-Frequency ECG for Detection of Myocardial Ischemia Associated with Right Coronary Artery Stenosis in IHD Patients

**DOI:** 10.17691/stm2020.12.1.11

**Published:** 2020

**Authors:** K.S. Kolosova, N.Yu. Grigoryeva, Yu.I. Kosyuga

**Affiliations:** PhD Student, Department of Faculty and Polyclinic Therapy, Privolzhsky Research Medical University, 10/1 Minin and Pozharsky Square, Nizhny Novgorod, 603005, Russia; Head of the Department of Faculty and Polyclinic Therapy, Privolzhsky Research Medical University, 10/1 Minin and Pozharsky Square, Nizhny Novgorod, 603005, Russia; Associate Professor, Department of Pathological Physiology, Privolzhsky Research Medical University, 10/1 Minin and Pozharsky Square, Nizhny Novgorod, 603005, Russia

**Keywords:** ischemic heart disease, myocardial ischemia, high-frequency ECG, hemodynamically significant stenosis

## Abstract

**The aim of the investigation** was to study the parameters of high-frequency ECG in leads V_1_, V_3_R, V_4_R, V_5_R, V_6_R as additional diagnostic criteria for detecting the areas of myocardial ischemia associated with stenosis of the right coronary artery (RCA) in IHD patients.

**Materials and Methods:**

The study involved 47 patients who underwent selective coronary angiography (SCA) for detection of IHD. The patients were divided into two groups based on the SCA results: group 1 included 28 patients with hemodynamically significant RCA stenosis; group 2 consisted of 19 patients with hemodynamically non-significant RCA stenosis. Prior to SCA, all patients underwent resting high-frequency ECG recording in 12 conventional leads and in leads V_3_R–V_6_R for 5 min. The study also involved 15 volunteers with no history of cardiovascular disease or IHD symptoms (control group) who underwent ECG in the same leads. The resulting data were processed and analyzed using the ArMaSoft-12-Cardio software (©ArMaSoft, 1995–2019, Russia), which made it possible to determine the presence or absence of reduced amplitude zones (RAZ) of the QRS complex in all morphological variants, the root-mean-square (RMS) deviation, and excess kurtosis.

**Results:**

Statistically significant differences in the RAZ parameter of the QRS complex were revealed in high-frequency ECG of patients with hemodynamically significant and non-significant RCA stenosis. The RAZ sum in leads V_1_, V_3_R, V_4_R, V_5_R, V_6_R was 7.86±0.77 and 3.58±0.53, respectively, while in patients with no IHD signs, it equaled 1.87±0.43 (p=0.00001).

The RMS value in patients with no IHD signs was 3.89±0.42, in patients with hemodynamically non-significant and significant RCA stenosis it equaled 3.51±0.34 and 2.73±0.24, respectively (p=0.008).

The kurtosis value was statistically significantly higher in patients with hemodynamically significant stenosis (1.07±0.12), in contrast to those with hemodynamically non-significant stenosis and without IHD (0.78±0.05 and 0.64±0.03, respectively).

An average correlation between the value of coronary stenosis and the sum of RAZ scores was found (r=0.66).

However, RMS and kurtosis parameters correlate with the degree of RCA stenosis at a lower level.

According to ROC analysis, the RAZ parameter showed better diagnostic results compared to RMS and kurtosis. Given the nonparametric nature of the available data, the prognostic capabilities of the studied parameters can be considered satisfactory as shown by the results of binary logistic regression.

**Conclusion:**

The RAZ parameter of high-frequency ECG in leads V_1_, V_3_R, V_4_R, V_5_R, V_6_R may serve as an additional diagnostic criterion for identifying the areas of myocardial ischemia associated with RCA stenosis in IHD patients.

## Introduction

Despite more than a hundred-year history of application, electrocardiography remains the single most important clinical test for the diagnosis of ischemic heart disease (IHD) [1]. ST segment changes in the electrocardiogram at rest and under physical stress serve as the conventional diagnostic criterion for myocardial ischemia, but the sensitivity of this parameter is low. Therefore, 24-hour Holter monitoring, exercise stress tests, and selective coronary angiography (SCA) are also used to diagnose myocardial ischemia. Exercise tolerance tests are known to have great diagnostic value, but they are impossible for patients with a number of diseases. 24-hour Holter monitoring reveals episodes of ischemia only in 10% of patients with asymptomatic ischemia. SCA is the gold standard in the diagnosis of IHD. However, this is an invasive diagnostic technique associated with certain economic costs and a number of restrictions.

Therefore, the development of new methods for detecting myocardial ischemia in IHD patients is a matter of great importance.

In recent decades, researchers have actively studied additional diagnostic ECG criteria based, in particular, on the analysis of the HF components of the QRS complex [2–4] using the high-frequency ECG method (HF ECG).

The interest of researchers in studying HF ECG is determined by the fact that this method has higher accuracy in the diagnosis of myocardial ischemia than conventional ECG. In 1986, the research group of Dr. S. Abboud was the first to find amplitude notches in the QRS complex of ECG signal when examining 3-lead HF ECG data. This phenomenon was called the reduced amplitude zone (RAZ) [5]. The researchers found the sensitivity of the RAZ criterion for IHD patients to be 75%. In paper [5], the average sensitivity of ST segment deviation criterion for the diagnosis of myocardial ischemia was shown to be 48±16% with an average specificity of 70±15%, while HF ECG data analysis provided 75±6% sensitivity with an average specificity of 80±6%. The HF ECG sensitivity for detecting latent IHD was 80%.

Later, other authors studied HF ECG. They recorded and analyzed 12 conventional leads instead of three, evaluated RMS and kurtosis criteria, developed a morphological classification of the RAZ parameter including Abboud RAZ (RAZ A), Abboud Percent RAZ (RAZ AP), NASA RAZ (RAZ N). In paper [6], the HF ECG sensitivity for detection of latent IHD was found to equal 75% [6], and in work [7] it was 68.8%.

It should be noted that all these results relate to the conventional 12-lead ECG system. However, in the heart there are zones whose electrical activity is not reflected in these leads. They are the region of the right ventricle of the heart and the posterobasal region of the left ventricle. In this regard, investigation of the HF ECG parameters in these areas may have important diagnostic value.

**The aim of the investigation** was to study the parameters of high-frequency ECG (RAZ, RMS, and kurtosis) in leads V_1_, V_3_R, V_4_R, V_5_R, V_6_R for identifying the areas of myocardial ischemia associated with stenosis of the right coronary artery (RCA) in IHD patients.

## Materials and Methods

The study involved 47 patients diagnosed with ischemic heart disease, admitted to City Clinical Hospital No.5 (Nizhny Novgorod) during 2017–2019 and referred for SCA (the main group), of which 16 were females (34%) and 31 were males (66%) aged 51 to 78 years (mean age was 63.1±6.3 years). The criteria of inclusion in the main group were considered to be the presence of IHD signs — stable angina pectoris of functional class II–III with chronic heart failure and preserved ejection fraction of functional class no higher than III [8–10]. Exclusion criteria were age younger than 18 and older than 90 years, congenital and acquired valvular heart disease, concomitant pathology in the acute stage, permanent atrial fibrillation, implanted electric cardiac pacemaker, dementia.

According to the SCA results, the patients of the main group were divided into two groups: group 1 included individuals with hemodynamically significant stenosis of the right coronary artery (n=28, 59.6%); group 2 comprised patients with hemodynamically non-significant stenosis (n=19, 40.4%). The control group consisted of 15 patients with no history of cardiovascular disease or IHD symptoms (group 3), of which 11 were females (73%) and 4 were males (27%) aged 39 to 50 years.

The study complies with the Declaration of Helsinki (2013) and was performed following approval by the Ethics Committee of Privolzhsky Research Medical University. Written informed consent was obtained from every patient.

Electrocardiogram recordings were made using a 12-channel computer-based electrocardiograph that provided registration of electrocardiographic signals in the frequency range 0.05 to 300 Hz.

Electrocardiograms were recorded in conventional 12 leads and in leads V_3_R, V_4_R, V_5_R, V_6_R immediately before the SCA procedure. The acquired electrocardiograms were analyzed using the ArMaSoft-12-Cardio software program (©ArMaSoft, 1995–2019, Russia). The following was performed during automatic analysis: formation of the averaged P–QRS–T complex for each of the recorded leads; measurement of all conventional amplitude-time parameters and calculated values; syndrome-based interpretation of the contour and rhythm; isolation of the high-frequency components of the QRS complex in the frequency range of 150–250 Hz; forming the envelope of the HF QRS components.

The reduced amplitude zones of the QRS complex were automatically identified by the program in each of the ECG leads and classified into the following categories based on their morphological characteristics: RAZ A (Abboud RAZ), RAZ AP (Abboud Percent), RAZ N (NASA RAZ) [5, 11].

The program assigned a specific score to each RAZ category: RAZ A — 1 score, RAZ AP — 2 scores, RAZ N — 3 scores. RMS (the root-mean-squared value of the high-frequency voltage potentials of the QRS complex) was automatically calculated for the time interval corresponding to the QRS complex duration.

The value of kurtosis parameter was also calculated (kurtosis is the distribution of deviations from the mean value of the HF QRS potentials) — a statistical estimate of the envelope signal in the QRS interval.

SCA was performed using the Innova 3 100 IQ X-ray unit (GE Medical Systems, 2011).

Analysis of the literature shows that currently there is no single classification for determining the severity of damage to the coronary arteries. According to the study carried out by Neeland et al. [12], all angiographic scales correlate well with each other. We have chosen the Coronary Artery Surgery Study (CASS) scale, where coronary artery narrowing ≥70% is considered as hemodynamically significant RCA stenosis [13]. It is the most convenient for assessing damage to one particular artery.

Statistical data processing was performed using IBM SPSS Statistics 24 (IBM), Statistica 10.0 for Windows (StatSoft) and Microsoft Office Excel 2016 (Microsoft) software. According to the research results, the mean values of the studied parameters were determined for the groups. Statistical comparison of the values was carried out using the Kruskal–Wallis test. The dependence between the selected parameters was assessed by correlation analysis. We used the methods of ROC analysis, binary logistic regression.

## Results and Discussion

The mean values of the HF ECG parameters studied in patients with hemodynamically significant and non-significant RCA stenosis and in individuals without IHD are shown in the Table.

**Table:** RAZ, RMS, and kurtosis values in leads V_1_, V_3_R, V_4_R, V_5_R, V_6_R in patients with hemodynamically significant and non-significant RCA stenosis and individuals without IHD (M±m)

HF QRS parameters (150–250 Hz)	Hemodynamically significant stenoses (n=28)	Hemodynamically non-significant stenoses (n=19)	Patients without IHD (n=15)	р
RAZ sum	7.86±0.77	3.58±0.53	1.87±0.46	0.00001
RMS	2.73±0.24	3.51±0.34	3.89±0.42	0.008
Kurtosis	1.07±0.12	0.78±0.05	0.64±0.03	0.0003

The calculations show that the sum of the RAZ scores in the specified leads has a moderate direct correlation with the RCA stenosis degree (r=0.66; p=0.0001). In these leads, the kurtosis parameter has a moderate direct correlation with the RCA stenosis degree (r=0.37; p=0.01). The RMS parameter shows a statistically insignificant weak negative correlation with the RCA stenosis degree (r=–0.29; p=0.053). The values of RAZ correlate with the stenosis degree stronger than other parameters, which may indicate its direct relation with narrowing of the RCA lumen.

Sensitivity, specificity and positive prognostic value were also calculated for all three HF ECG parameters in the study.

For the RAZ value, sensitivity was 75% (95% CI — 0.62–0.85), specificity was 68.4% (95% CI — 0.49–0.84), positive prognostic value (PPV) — 77.8% (95% CI — 0.64–0.89).

For the RMS value, sensitivity was 89.3% (95% CI — 0.8–0.97), specificity was 21.1% (95% CI — 0.08–0.32), PPV — 62.5% (95% CI — 0.56–0.68).

For kurtosis value, sensitivity was 78.6% (95% CI — 0.66–0.89), specificity was 52.6% (95% CI — 0.34–0.68), PPV — 71% (95% CI — 0.59–0.81).

According to the ROC analysis, the area under the curve (AUC) for the RAZ parameter was 0.82 (95% CI — 0.7–0.94; p=0.0001), for the RMS parameter — 0.311 (95% CI — 0.16–0.47; p=0.029), for the kurtosis parameter — 0.70 (95% CI — 0.55–0.86; p=0.019). The RMS parameter has low diagnostic value for revealing the hemodynamic significance of RCA stenosis, while the higher values of the area under the ROC curve in the RAZ parameter show its greatest diagnostic value (see the Figure).

**Figure:**
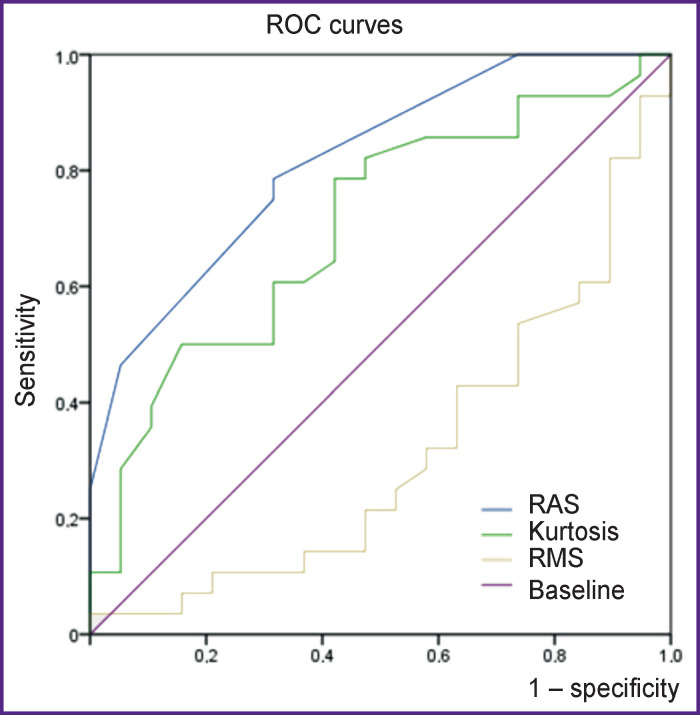
Comparison of ROC analysis results for RAZ, RMS, and kurtosis parameters to determine the degree of right coronary artery stenosis

The applied binary logistic analysis showed that the odds ratio for the RAZ parameter was 1.634 (95% CI — 1.5–2.3; p=0.007), for the RMS parameter — 0.514 (95% CI — 0.27–0.98; p=0.043), for the kurtosis parameter — 8.6 (95% CI — 0.31–239.31; p=0.205). Thus, the probability of obtaining increased RAZ values in patients with hemodynamically significant RCA stenosis is 1.634 times higher than in patients with hemodynamically non-significant stenosis. According to binary logistic analysis, the RMS and kurtosis parameters appeared to be non-diagnostic.

To assess the proportion of observations identified correctly (pre-diagnosed by applying the program), the concordance coefficient is used. Overall, our proposed technology interprets 74.5% of cases correctly: 73.7% of the total number of hemodynamically non-significant stenosis cases and 75.0% of hemodynamically significant stenoses were predicted correctly. The value of χ^2^ was 22.44 at p=0.0001.

## Conclusion

Analysis of the obtained data shows significant differences between the RAZ parameters in patients with hemodynamically significant and hemodynamically non-significant stenosis of the right coronary artery. A significantly higher mean value of the total RAZ score is observed in leads V_1_, V_3_R, V_4_R, V_5_R, V_6_R in the group with hemodynamically significant stenosis as compared to patients with hemodynamically non-significant stenosis and individuals without IHD.

The RMS parameter has a higher value in individuals without IHD and patients with hemodynamically non-significant stenosis as compared to patients with hemodynamically significant stenosis of the right coronary artery.

The kurtosis parameter has significant differences between patients in all three groups.

According to ROC analysis, the RAZ parameter shows better results compared to the RMS and kurtosis parameters.

Given the nonparametric nature of the available data, the prognostic capabilities of the studied parameters can be considered satisfactory as demonstrated by the results of binary logistic regression.

The diagnostic value of the RAZ parameter has also been proved by the correlation method, diagnostic sensitivity and specificity data.

Therefore, the RAZ parameter of high-frequency ECG in leads V_1_, V_3_R, V_4_R, V_5_R, V_6_R may serve as an additional diagnostic criterion for detecting the areas of myocardial ischemia associated with stenosis of the right coronary artery in IHD patients. It is useful for screening and diagnosis in conditions of limited time. The method is low-cost and easy to implement.

A patent application dated May 29, 2019 was filed in accordance with the investigation results.
